# Emotion expression and cooperation under collective risks

**DOI:** 10.1016/j.isci.2023.108063

**Published:** 2023-09-29

**Authors:** Celso M. de Melo, Francisco C. Santos, Kazunori Terada

**Affiliations:** 1 DEVCOM U.S. Army Research Laboratory, Playa Vista, CA 90094, USA; 2 INESC-ID and Instituto Superior Técnico, Universidade de Lisboa, IST-Taguspark, Porto Salvo 2744-016, Portugal; 3 Gifu University, 1-1 Yanagido, Gifu 501-1193, Japan

**Keywords:** Social sciences, Psychology

## Abstract

The difficulties associated with solving Humanity’s major global challenges have increasingly led world leaders and everyday citizens to publicly adopt strong emotional responses, with either mixed or unknown impacts on others' actions. Here, we present two experiments showing that non-verbal emotional expressions in group interactions play a critical role in determining how individuals behave when contributing to public goods entailing future and uncertain returns. Participants' investments were not only shaped by emotional expressions but also enhanced by anger when compared with joy. Our results suggest that global coordination may benefit from interaction in which emotion expressions can be paramount.

## Highlights

• Role of emotion expression in collective risk dilemmas has been neglected
• Participants engaged with group that expressed emotion reflecting high or low demand
• Participants invested more when expressions reflected high demand
• We show emotion expression can help solve risky collective societal dilemmas

## Graphical abstract

**Figure:**
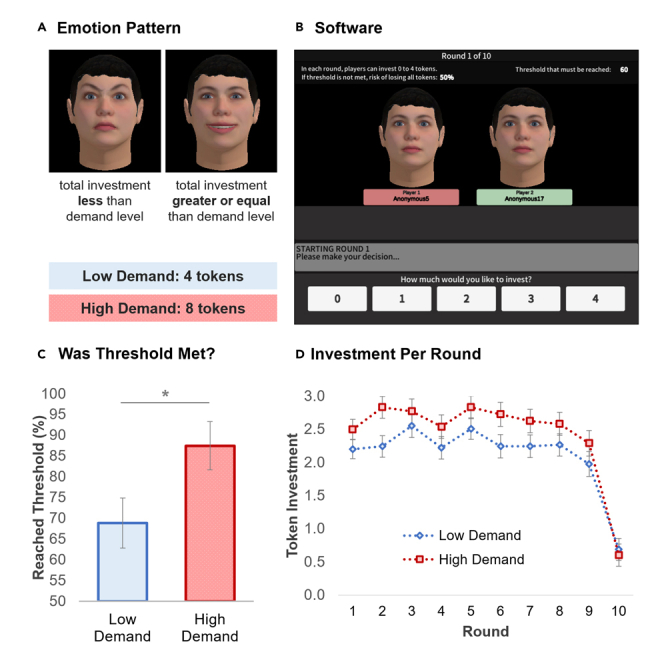

## Introduction

Several human endeavors can be abstracted as public good games wherein individual and collective interests conflict. Participants can contribute to a common good, but all stand to benefit from it independently of their contribution, hence the social dilemma. Collective risk dilemmas (CRDs)^1^^,^^2^ are an important variation where participants, who are given an initial endowment, have multiple rounds to reach a contribution threshold. If the threshold is not met, all participants risk a disaster (losing all their endowment). This creates a loss-avoidance game characterized by non-linear, time-delayed, and uncertain returns, which has been used to model the critical global challenge of climate change^1^^,^^2^^,^^3^^,^^4^ and many other socioeconomic dilemmas^5^^,^^6^^,^^7^^,^^8^ involving prospective choices. Due to its broad practical relevance, researchers have studied how structural aspects of the game—such as risk of failure,^1^^,^^2^ group size,^2^^,^^9^ timing uncertainty,^10^ anonymity conditions,^11^ and wealth inequality^12^^,^^13^—shape decision-making in these situations. Building on earlier findings^14^ in the context of other social dilemmas, researchers have also found that verbal communication^12^^,^^15^^,^^16^ can promote cooperation in the CRD, for instance, through pledges or by reporting defection. However, considerably less attention has been given to nonverbal communication, particularly the influence of emotion expressions on collective action.

There is increasing evidence that emotion expressions serve critical social functions and influence others' decision-making.^17^ Emotion is generally thought to arise from continuous appraisal of events with respect to the individual’s beliefs and goals.^18^ Emotion, thus, is event-focused and is relatively intense and of short duration, in contrast to other affective phenomena such as mood. Different patterns of appraisals lead to different emotions with concomitant physiological (e.g., changes to the peripheral nervous system), cognitive (e.g., appraisals), motivational (e.g., resulting action tendency), and expressive responses (e.g., facial displays). However, emotion expressions do not always necessarily reflect the internal emotional state, as people often up- and down-regulate expression for social objectives (e.g., masking anger with a polite smile) and may express them purely for social goals (e.g., joy to convey affiliative intent).^17^^,^^19^ In fact, whether expressions reflect internal emotion may be less important as, often, it serves the same social purpose—for instance, if someone expresses anger, it may be irrelevant whether the individual is truly experiencing anger or simply conveying to others that one wants the current state of affairs to change, because the social consequence is the same. Furthermore, emotion expressions are a particularly effective way to achieve this social function as they are often perceived to be a more authentic signal,^20^ when compared with others.^21^ Several social functions of emotion have now been identified^22^^,^^23^^,^^24^^,^^25^^,^^26^ and, of particular interest here, is communicating mental states and intentions to others,^27^^,^^28^ which subsequently shape people’s decisions.^29^

Which social functions, then, can emotion expressions serve in CRDs? Here we focus on two pervasive emotions—joy and anger—which commonly lead to opposite consequences in others. Joy often serves an affiliation function, contributing to fostering interpersonal relationships,^30^ positively shaping other’s appraisals of a situation,^31^^,^^32^ and helping construe a group setting as cooperative.^33^ However, joy can also have counterproductive effects. In negotiation, expressions of joy were perceived to indicate that the expresser had low aspirations and led to higher demand and exploitative behavior from others.^34^ Anger, in contrast, often serves a dominance function, communicating unsatisfaction with the current situation and blame to others. Anger, thus, can instigate change. In negotiation, expressions of anger led others to infer high aspirations from the expressers and, consequently, motivate others to make higher concessions;^34^ however, anger can also impose a cost in the relationship, which may lead to longer term negative consequences.^35^ In groups, expressions of anger can signal social norm violation^36^ and encourage deviant members to conform to the majority.^37^^,^^38^ In addition, the effects of emotion expressions are contingent on the perceived appropriateness of the expression.^17^^,^^39^^,^^40^ For instance, in cooperative settings the expression of anger can lead to negative consequences, in contrast to settings where there are conflicts of interest.^41^ Nevertheless, despite this growing literature on the social effects of emotion, to the best of our knowledge, research on the effects of emotion expression in complex N-player dilemmas, such as CRDs, is still missing.

This work, thus, builds on prior work showing that emotion expressions are pervasive and play a central role in social decision-making,^17^ such as negotiation and social dilemma settings, and even in situations involving interactions with machines.^42^ The scope of this paper, given this context, is to identify specific social functions of emotional expressions in CRDs (but, see the limitations of the study section for follow-up work to further understand how pervasive emotion expressions are specifically in CRDs). For this purpose, we focus on prior work in negotiation where emotion expressions have been shown to occur frequently (in laboratory and “in the wild” settings) and often communicate the individual’s aspirations and demand level^17^^,^^34^ (however, see the Discussion for important differences between negotiation and CRDs). Our central research question, therefore, is: can emotion expressions communicate demand level and shape decision-making in CRDs? Our hypothesis is that when emotion expressions convey high demand, participants will invest more in CRDs; in contrast, when emotion expressions convey low demand, participants will invest less in CRDs.

We present two experiments where participants engaged in a CRD^1^ with counterparts that portrayed emotional profiles communicating different demand level. In the first experiment, we show that non-verbal emotional expressions in group interactions shaped individual behavior in CRDs. In the second experiment, we replicate this effect, with a new sample from a different participant pool, and provide insight on the mechanism driving this effect.

## Results

### Experiment 1: do emotion expressions shape decision-making?

Participants engaged in groups composed of three players for multiple rounds (10 rounds). In each round, they could contribute part (0–4 tokens) of an initial endowment (40 tokens). Tokens were important as they would enter a lottery for $30, and thus, the more tokens the participant kept, the higher the chances of winning the lottery. Tokens invested in the common pool were discarded. A collective target was reached if a minimum number of contributions was achieved (60 tokens) over multiple rounds. In that case, everyone kept whatever they had at the end of the game. However, failure to reach this threshold implied that all endowments were at risk of being lost. All members would lose their remaining endowments with a probability measuring the risk of collective disaster, which in our experiment was 50%. At this risk level, the average gain for a free rider—who offers nothing—is the same as for a fair sharer—who invests the fair amount at each round.^1^ This setting, thus, is optimal for studying social factors, such as emotion expressions, and their role in mitigating free riding.

**Figure 1:**
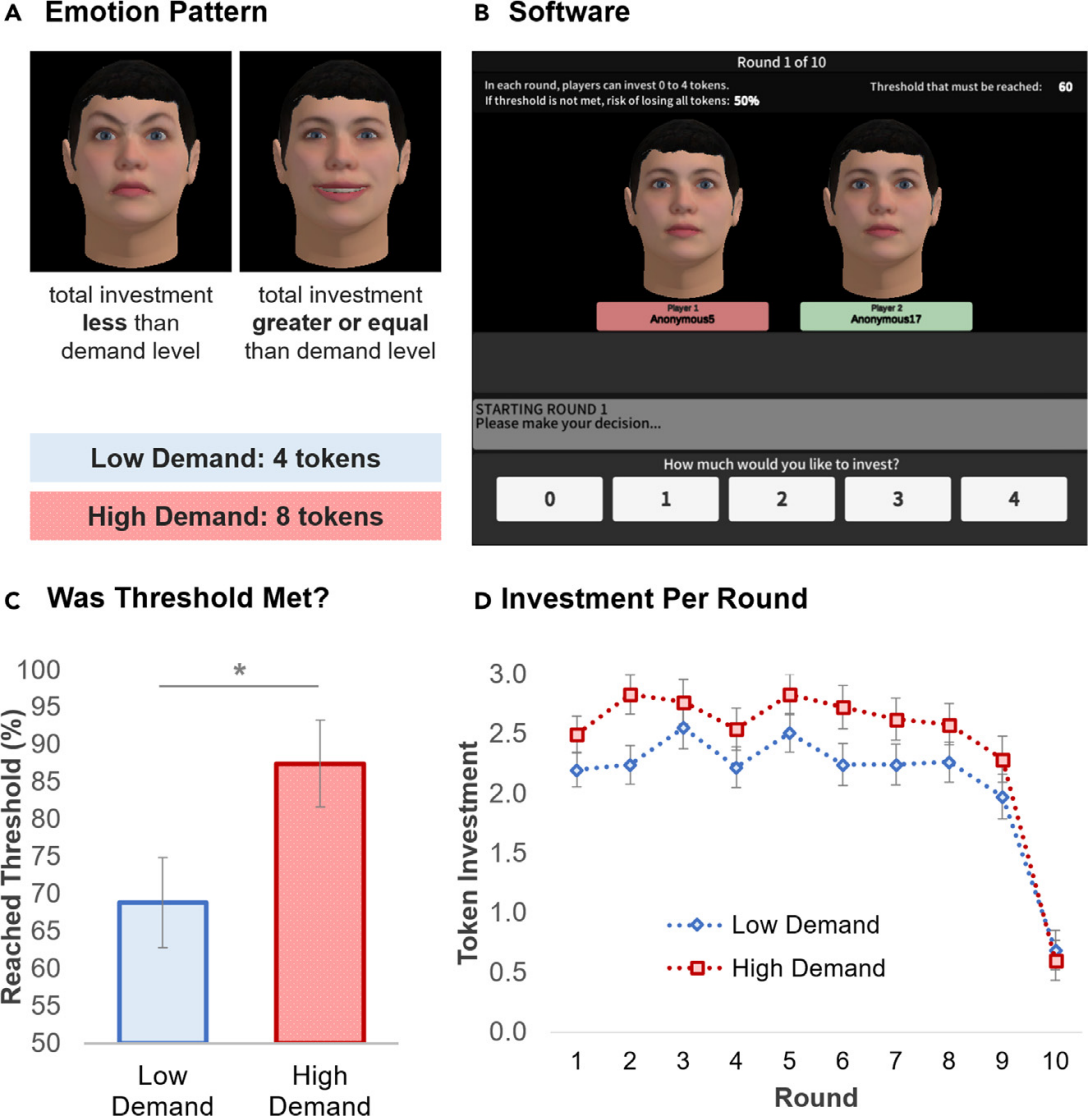
Participants reached the threshold and invested more in the collective risk dilemma when the group showed emotion reflecting high demand (Experiment 1) (A) The emotion expression pattern reflected a (low or high) demand level. When the total investment was below the demand level, the group showed anger; otherwise, joy. (B) The collective risk dilemma software. (C) Participants reached the investment threshold more often with the high-demand group. (D) Investment per round. ∗p < 0.05. Error bars show standard errors.

To enhance experimental control, participants were instructed they would engage with other participants but, in fact, the counterparts were scripted; similar methods have been used in other studies of human decision behavior (e.g.,^27^^,^^29^^,^^34^). Our prior work also suggests that most participants are not aware that they are engaging with scripted counterparts^27^^,^^29^ (prior to being debriefed) but, even for those who are, the social effects of emotion expression tend to follow a similar pattern.^43^ The counterparts’ investment was fixed and designed to total the fair amount (i.e., 40 tokens): (4, 0), (1, 2), (2, 1), (3, 3), (0, 3), (2, 1), (0, 4), (3, 2), (3, 4), (2, 0). Therefore, to meet the threshold, the participant had to invest at least 20 tokens total or an average of 2 tokens per round—i.e., the fair amount. The design, therefore, allowed us to study whether emotion would encourage or discourage participants to invest the total fair amount, with corresponding implications to reaching the critical threshold. The emotion expressions were designed to serve the social function of communicating information about demand level concerning the investments of their peers. When the total investment was below the demand level, preprogrammed artificial counterparts showed anger; otherwise, counterparts would portray joy (Figure 1A) (see the supplemental information (SI) for a pilot study that emphasizes the importance of having contingent emotion expressions). To add a small amount of noise to the design, in rare occasions, one of the counterparts would show the neutral expression instead of the emotional expression. There were two experimental conditions: low demand (4 tokens) and high demand (8 tokens). Notice that the fair demand level would have been 6 tokens. Moreover, we note that “demand level” was never explicitly mentioned in the instructions or during the task, and thus, the only source of information participants had about it was the emotional expression.

To convey emotion expression, all players were represented in the task by a virtual face that was able to express emotion (Figure 1A). There are several advantages to using virtual agents, or avatars, to study human behavior, such as increased experimental control.^44^ Participants were instructed that the avatar is how others would see them and, moreover, they would have the opportunity to self-report how they felt at the end of each round. Thus, the design of the task was perfectly symmetrical, with all participants having their own avatar that could express emotion. To avoid any potential bias, we used the same virtual face for all participants,^45^ but with different colors for the labels (Figure 1B). The expressions of joy and anger correspond to prototypical configurations of the facial muscles.^46^ These expressions were validated, with independent participant samples, elsewhere.^27^^,^^29^^,^^45^ Further details on the methods are available in the STAR Methods section and a video showing the experimental software in the SI (Video S1).

Video S1. Software used in experimental studies

We recruited participants (n = 93) from Mechanical Turk, an online pool commonly used to study human decision-making behavior (e.g.,^27^^,^^29^) (see STAR Methods for further details on the sample). The main result was that the CRD threshold was achieved more often with the high-demand than the low-demand group (Figure 1C, Wilcoxon *W* = 1914.00, *Z* = −2.17, p = 0.030). Further supporting this effect, when looking at the investment across rounds (Figure 1D), a Round × Emotion mixed analysis of variance (ANOVA) confirmed that participants invested more with the high-demand (*M* = 2.43, *SE* = 0.103) than the low-demand group [*M* = 2.12, *SE* = 0.106; *F*(1, 91) = 4.57, p = 0.035, $\eta_{p}^{2}$ = 0.048].

### Experiment 2: what is driving the effect?

In a second experiment, we sought to (1) replicate the emotion expression effect with a separate sample (from a different online pool, Prolific), (2) introduce a control/no-emotion condition for comparison, and (3) gather insight on the mechanism driving the effect by analyzing participants’ self-reports of emotion experience and several post-task questionnaires. The experiment followed the same procedure as Experiment 1, except that in this case there were three conditions: low demand versus no emotion versus high demand. In the control/no-emotion condition, the group’s avatars always showed the neutral expression. We recruited participants (n = 283) from another common online pool, Prolific (see STAR Methods for further details on the sample).

**Figure 2:**
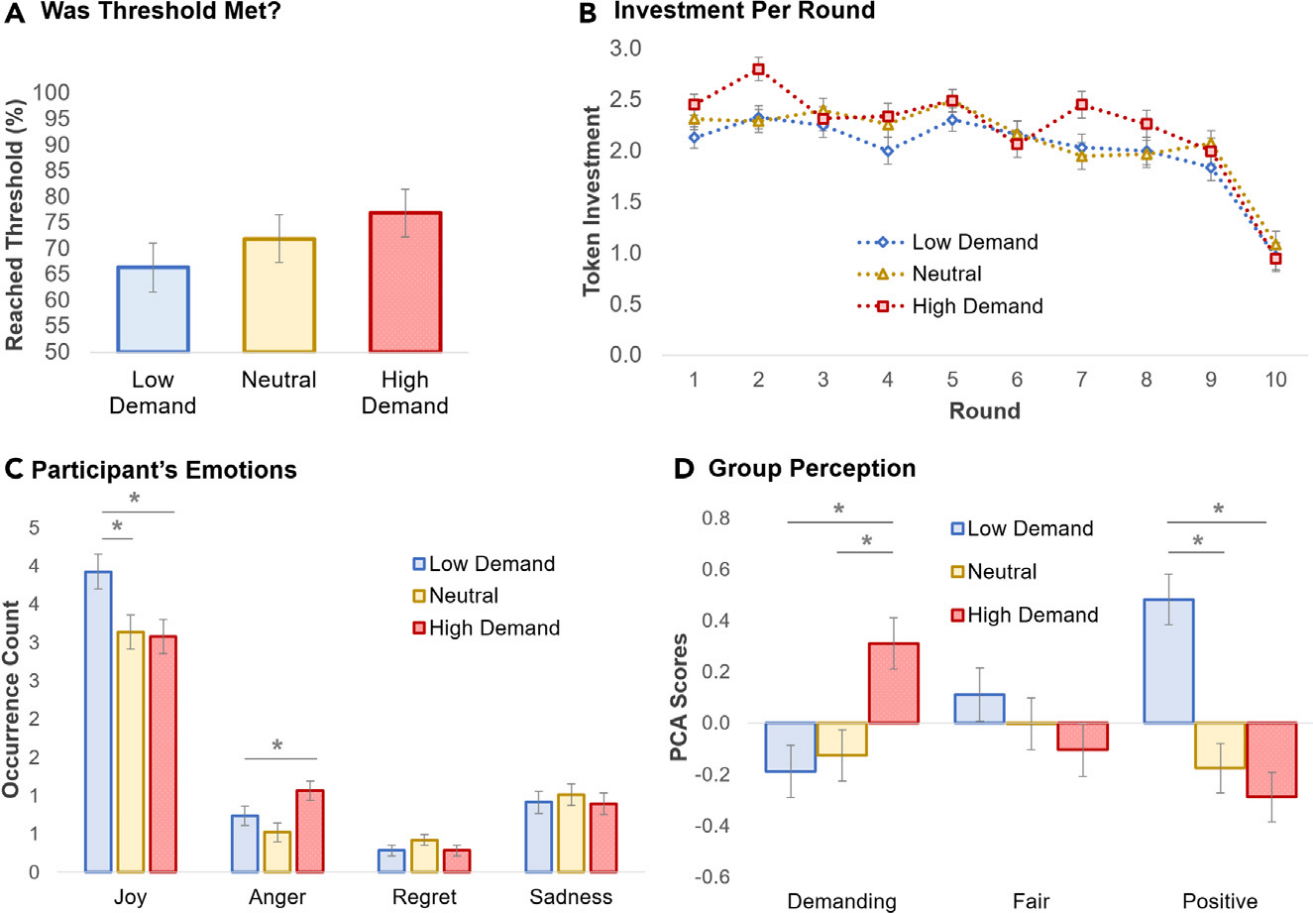
Participants perceived the high-demand group to be the least positive and most demanding (Experiment 2) (A) Participants were more likely to reach the threshold with the high-demand group and the least likely with the low-demand group. (B) Investment per round. (C) Participants self-reported the most joy with the low-demand group and the most anger with the high-demand group. (D) The high-demand group was perceived to be the least positive and most demanding, whereas the low-demand group was perceived to be the happiest with the offers. ∗p < 0.05. Error bars show standard errors.

The results showed a similar trend as in Experiment 1, with participants tending to reach the threshold more often in the high-demand condition (Figure 2A, Kruskal-Wallis *H* = 2.55, p = 0.279). This trend was stronger when, as in Experiment 1, we consider only the low- and high-demand conditions: Wilcoxon *W* = 8187.50, *Z* = −1.59, p = 0.111). The effect may have been less strong when compared with the previous experiment due to higher variance in this subject pool and overestimation of the effect size, which resulted in a low estimate for the participant sample size (see STAR Methods for details on sample size estimation). Nevertheless, a Round × Emotion mixed ANOVA replicated the main effect of emotion in investment level (Figure 2B, *F*(2, 280) = 3.24, p = 0.041, $\eta_{p}^{2}$ = 0.023), with participants investing more with the high-demand than the low-demand group.

One of the objectives for this experiment was to get insight on these effects. To accomplish this, we first asked participants to report, throughout the task after each round outcome was shown, how they felt about the outcome (Figure 2C). For each emotion, we ran an ANOVA on the number of times the emotion was experienced during the task (with Bonferroni corrections for pairwise comparisons), which revealed that participants self-reported experiencing more joy with the low-demand than the neutral and high-demand groups [*F*(2, 280) = 4.45, p = 0.012, $\eta_{p}^{2}$ = 0.031; low demand versus neutral: p = 0.040; low demand versus high demand: p = 0.023] and more anger with the high-demand than the low-demand group [*F*(2, 280) = 4.98, p = 0.007, $\eta_{p}^{2}$ = 0.034; high demand versus low demand: p = 0.06]. There were no statistically significant effects for regret and sadness.

Further insight comes from analyzing participants’ subjective impressions of the group. We asked several questions following the task and ran a principal component analysis with varimax rotation on these questions, which resulted in three factors, shown in Figure 2D, characterizing the group as demanding (e.g., “The group was very demanding”), fair (e.g., “The group wanted all to contribute their fair share”), and positive (e.g., “Was the group hostile or friendly?”) (see Table S1 in the SI for more details, including all loadings). We ran ANOVAs on these factors (with Bonferroni corrections for pairwise comparisons), which revealed a main effect of perceptions of demand, with participants perceiving, as expected, the high-demand group to be more demanding than the neutral and low-demand groups [*F*(2, 280) = 7.30, p = 0.001, $\eta_{p}^{2}$ = 0.050; high demand versus neutral: p = 0.007; high demand versus low demand: p = 0.002]. On the other hand, the low-demand group was perceived to be more positive than the neutral and high-demand groups [*F*(2, 280) = 18.16, p < 0.001, $\eta_{p}^{2}$ = 0.115; low demand versus neutral: p < 0.001; low demand versus high demand: p < 0.001]. There was no statistically significant effect for perceptions of fairness [*F*(2, 280) = 1.09, p = 0.337].

## Discussion

The collective risk dilemma captures a complex social situation where avoiding a catastrophe requires the individual to sacrifice self-interest for the common objective. In our setting, the expected outcome for a free rider was the same as for a fair sharer, yet emotion expressions shaped participants’ investments. Participants in the high-demand group, who often experienced anger, were likely to invest more than participants in the low-demand group, who often experienced joy. Moreover, this effect was replicated with two independent samples from two distinct participant pools. Importantly, those in the high-demand group were also more likely to reach the investment threshold. Seemingly paradoxically, anger, a negatively valenced emotion had a more beneficial effect than joy, a positively valenced emotion. The result, thus, emphasizes the importance of context in understanding the behavioral consequences of emotion expressions. It is not the emotion per se that matters, but what it means.^27^

What, then, explains the social effects of joy and anger in the CRD? Our results suggest that anger communicated that the group had high aspirations and was demanding more from their members, whereas joy signaled satisfaction with the current state of affairs. In fact, the emotion patterns were designed to serve the social function of communicating demand level; this aligns with findings in the negotiation literature showing that anger led to higher concessions than joy, as it reflects higher aspirations from the expressers.^34^ Our results, furthermore, did not provide evidence for the alternative explanation that anger communicated failure to abide to the prevailing group’s social norms,^36^ which in this case may have been that everyone should cooperate and contribute their fair share,^47^ thus persuading deviant group members to conform to the group’s majority.^37^^,^^38^ Our subjective measures did not suggest any difference in perception of fairness between the low- and high-demand groups. Further study is, nevertheless, needed on the role of social norms in shaping decision and expressive behavior in CRDs.

The results also suggest potential limits to the social effects of emotion in CRDs. Even though expressing emotion that communicates high demand led to increased likelihood of reaching the threshold, the subjective measures suggest that the high-demand group was perceived as being less positive than the low-demand group. This is in line with findings showing that anger led to longer term costs in negotiation, despite short-term rewards.^35^ It is important, nevertheless, to distinguish negotiation and CRDs: in CRDs, unlike in negotiation, any individual concession is not to the exclusive benefit of others but for the collective interest, thus potentially mitigating these longer-term costs. Therefore, although the current work suggests that practitioners should resort to anger to encourage investment in CRDs, this should be paired with appropriate action aimed at preserving the relationship and mitigating longer term costs. Future work is further needed to understand the tradeoffs between short- and longer term consequences of emotion expression in CRDs.

The results may also inform extensions to existent theoretical models of human behavior. Although several cooperation mechanisms have been identified that explain why people make decisions that take others into consideration,^48^ as is needed to succeed in CRDs, emotion expressions are notably absent in these models. Some work has considered the role of experienced emotion on emergence of cooperation,^49^ but much less attention has been given to the role of emotion *signals* in shaping others’ decisions. The present work, in fact, aligns nicely with a growing body of work emphasizing the influence of emotion expression in shaping cooperation,^17^^,^^20^^,^^27^ moral preferences,^50^^,^^51^ and social norms.^29^

Our work sheds light on an important social dimension missing in the study of management of collective risks: the role of emotion expression. Our results suggest that emotions can provide a route for cooperation to thrive in situations where collective returns are only achievable in the future, such as in the case of the mitigation of environmental problems. Our findings emphasize the potential practical benefits of (direct and virtual) interaction in which emotion expressions play a prominent role and the need for communication channels with sufficient bandwidth for the expression of nonverbal cues. This is in line with prior literature indicating that nonverbal behavior can help build rapport with others^17^ and help clarify intent to others^27^ in negotiation and dyadic social dilemmas. The results also hint at the value of virtual (emotional) avatars in establishing cooperation when humans engage with each other in immersive settings.^52^ In an era of human interactions shaped by technological artifacts, our results indicate how such instruments may sway human coordination by allowing (or not) the broadcast of emotional cues. Overall, by regulating, understanding, and expressing appropriate emotion, it can be possible to alter group behavior, promote collective action, and avoid the catastrophic consequences of failing to resolve risky societal dilemmas.

### Limitations of the study

Our results show that emotion expressions can shape behavior in CRDs by communicating demand level to others; however, the present work does not explicitly address the question of how pervasive is emotional expression in CRDs. Building on prior work in negotiation and social dilemmas,^17^ it may be plausible to assume that is the case but, nevertheless, it is worth following-up with experiments directly focusing on this important aspect. These experiments could, for instance, measure facial expressions in face-to-face CRD interactions or analyze public data that may be available for CRD-like settings.

To further understand the causal mechanisms underlying the effects reported here, it would also be interesting to complement the present study with more experiments, involving larger sample sizes, that explicitly measure perceived demand, fairness, and relevant social norms immediately following the expression of emotion, rather than post-task as we did here. In addition, we currently rely on self-reported emotion expressions to assess participants’ emotions throughout the task; however, with this approach it becomes harder to distinguish premeditated from genuine emotion expression. Although people often express emotion that does not necessarily reflect their true internal emotional state for social reasons,^17^^,^^19^ it may be, nevertheless, interesting to measure facial expression and physiology directly to better assess the participants’ internal emotional state. These metrics could then be subjected to a multiple mediation analysis to better assess the factors mediating the effect of emotion expression on investment in CRDs.

Finally, as noted in the Introduction, emotion expressions are a particularly informative signal as they often occur automatically (independently of whether they reflect true emotional state) and, consequently, have been shown to be reliable for inferences about others’ intentions in social decision-making.^20^ Still, it would be interesting to explicitly compare facial expressions of emotion to other forms of communication (e.g., verbal statements about aspirations or demand level), to better appreciate the relevance of emotional expressions in CRDs.

## STAR★Methods

### Key resources table

**Table:** 

REAGENT or RESOURCE	SOURCE	IDENTIFIER
**Deposited data**		

Experimental data	This paper. Available in SI	

### Resource availability

#### Lead contact

Further information and requests for resources should be directed to Kazunori Terada (terada@gifu-u.ac.jp).

#### Materials availability

This study did not generate any new materials.

### Experimental model and study participant details

All experimental methods were approved by the Medical Review Board of Gifu University Graduate School of Medicine (IRB ID#2018-159). As recommended by the IRB, written informed consent was provided by choosing one of two options in the online form: 1) “I am indicating that I have read the information in the instructions for participating in this research and have had a chance to ask any questions I have about the study. I consent to participate in this research.”, or 2) “I do not consent to participate in this research.” All participants gave informed consent and, at the end, were debriefed about the experimental procedures. All the experiment protocols involving human subjects was in accordance to guidelines of the Declaration of Helsinki.

#### Study 1

For Experiment 1, participants for the experiment were recruited from Amazon Mechanical Turk. All participants were from the United States and had an approval rate, based on prior work in this pool, of at least 95%. To estimate the sample, we followed the power calculations proposed by Jacob Cohen and implemented in G∗Power [https://www.psychologie.hhu.de/arbeitsgruppen/allgemeine-psychologie-und-arbeitspsychologie/gpower.html (Last accessed: March-8, 2023)]. We estimated sample size for a between-participants design: low demand vs. high demand. Based on our prior studies, we estimated a medium effect size (Cohen’s *d* = 0.5). For α = .05 and statistical power of 0.80, the recommended total sample size was 102 participants. When recruiting from this pool, it is common for some participants to fail to successfully complete the task or otherwise make data entry errors. In practice, we ended up with a sample of 93 participants with the following demographics: 64.5% males; age distribution (18 to 21 years, 1.1%; 22 to 34 years, 29.0%; 35 to 44 years, 46.2%; 45 to 54 years, 16.1%; 55 to 64 years, 6.5%; over 64 years, 1.1%).

#### Study 2

For Experiment 2, participants were recruited from Prolific. All participants were from the United States and had an approval rate, based on prior work in this pool, of at least 95%. We estimated sample size for a between-participants design: low demand vs. control vs. high demand. Based on Experiment 1, we estimated a small to medium effect size (*f* = 0.2). For α = .05 and statistical power of 0.95, the recommended total sample size was 321 participants. When recruiting from this pool, it is common for some participants to fail to successfully complete the task or otherwise make data entry errors. In practice, we ended up with a sample of 283 participants with the following demographics: 48.4% males; age distribution (18 to 21 years, 3.9%; 22 to 34 years, 41.7%; 35 to 44 years, 24.7%; 45 to 54 years, 12.7%; 55 to 64 years, 11.0%; over 64 years, 6.0%). As noted in the main text, in the context of only replicating a trend for the effect on reaching the threshold, in retrospect, it would likely have been more appropriate to assume a small effect size (*f* = 0.15), which would have resulted in a larger estimate for this participant sample size.

#### Ethics declarations

All experimental methods were approved by the Medical Review Board of Gifu University Graduate School of Medicine (IRB ID#2018-159). As recommended by the IRB, written informed consent was provided by choosing one of two options in the online form: 1) “I am indicating that I have read the information in the instructions for participating in this research and have had a chance to ask any questions I have about the study. I consent to participate in this research.”, or 2) “I do not consent to participate in this research.” All participants gave informed consent and, at the end, were debriefed about the experimental procedures. All the experiment protocols involving human subjects was in accordance to guidelines of the Declaration of Helsinki.

### Method details

The experiments were fully anonymous for participants. To accomplish this, players had anonymous names, and we never collected any information that could identify participants. To preserve anonymity with respect to experimenters, we relied on the anonymity system of the online pools we used. When interacting with participants, researchers are never able to identify the participants, unless we explicitly ask for information that can identify them (e.g., name, email, or photo), which we did not.

#### Study 1

Participants engaged in the CRD in groups composed of three players for multiple rounds (10 rounds). The counterparts’ investment was fixed and designed to total the fair amount (i.e., 40 tokens). To convey emotion expression, all players were represented in the task by a virtual face which was able to express emotion reflecting low or high demand. Participants were paid $2.50 for participating in the experiment, which is a typical amount for Amazon Mechanical Turk for a 20-minute experiment at the time (April 2022). Moreover, they could earn more money according to their performance in the task. Each token earned in the task – i.e., that was not invested in the common pool – was automatically converted to a ticket for a lottery worth $30.

#### Study 2

The experiment followed the same procedure as Experiment 1, except that in this case there were three conditions: low demand vs. no emotion vs. high demand. In the control no emotion condition, the group’s avatars always showed the neutral expression. Participants were paid $4.00 for participation, which is a typical amount for Prolific for a 20-minute experiment at the time (January 2023). Each token earned in the task was also converted to a ticket for a $30 lottery.

### Quantification and statistical analysis

#### Study 1

To compare threshold agreement between the two conditions, we ran a Wilcoxon test. To compare investment, we ran a Round × Emotion mixed ANOVA.

#### Study 2

To compare threshold agreement between the three conditions, we ran a Kruskal-Wallis test. To compare investment, we ran a Round × Emotion mixed ANOVA. To analyze subjective measures, we ran factorial ANOVAs.

## Data Availability

Experimental data for the two studies in this paper are available in the SI. No original code created for this paper.

## Supplemental information

Document S1. Figures S1–S3 and Table S1

Data S1. Experimental data for Study 1

Data S2. Experimental data for Study 2
