# Supplementary material for: Emotion expression and cooperation under collective risks
Source: iScience. 2023 Sep 29;26(11):108063. doi: 10.1016/j.isci.2023.108063 (PMC10616387; doi:10.1016/j.isci.2023.108063)
Supplement: Document S1. Figures S1–S3 and Table S1 [file mmc1.pdf]

iScience, Volume *26*

Supplemental information

Emotion expression and cooperation under collective risks

Celso M. de Melo, Francisco C. Santos, and Kazunori Terada

Supplemental Information

Emotion Expression and Cooperation under Collective Risks

Celso M. de Melo, Francisco C. Santos, Kazunori Terada

Pilot Study with Non-Contingent Emotion Expressions

Prior to running the experiments reported in the main text, we conducted a pilot study where participants engaged with counterparts that expressed emotion that was not contingent on the total round investment: in the *anger* condition, the counterparts would always show anger (even if the total investment was high); in the *joy* condition, the counterparts would always show joy (even if the total investment was low). We report this pilot to provide insight on the importance of having contingent emotion expression to achieve the effects reported in this paper. We recruited a sample of 94 participants from Amazon Mechanical Turk for this study. We ran a Round × Emotion ANOVA, which did *not* show a significant main effect of emotion ($F(1, 92) = 0.82$, $p = 0.816$). The results suggest that non-contingent expression of anger is a noisier communication signal that is harder to decode by participants; analogously for non-contingent expression of joy. Following this outcome, we adjusted our experimental design to follow contingent emotion expression as described in the main text.

Investment Analysis Following Specific Emotion Expressions

For additional detailed insight on the effects described in the main text, we calculated average investment following each type of emotion expression for the low demand and high demand conditions (we ignored the control condition for Experiment 2 in this analysis). We ran mixed ANOVAs which, as shown in Figure S1, revealed statistically significant interactions between the emotion condition and the group's emotion expression in the prior round – Experiment 1: $F(1, 91) = 122.04$, $p < 0.001$, $\eta_p^2 = 0.573$; Experiment 2: $F(1, 185) = 137.36$, $p < 0.001$, $\eta_p^2 = 0.426$. Following expressions of joy, there was no difference in investment between low vs. high demand groups; however, following expressions of anger, participants were likely to invest more in the high demand than the low demand condition. This may suggest that the effect of emotion is contingent on the perceived appropriateness of

the expression to the situation. Prior work, similarly, indicates that anger can lead to positive change in competitive interaction, but be counterproductive in cooperative settings. Other studies have also suggested an important moderating role of culture on the effects of emotion, with different cultures having different expectations about the appropriateness of emotion expression in social settings.

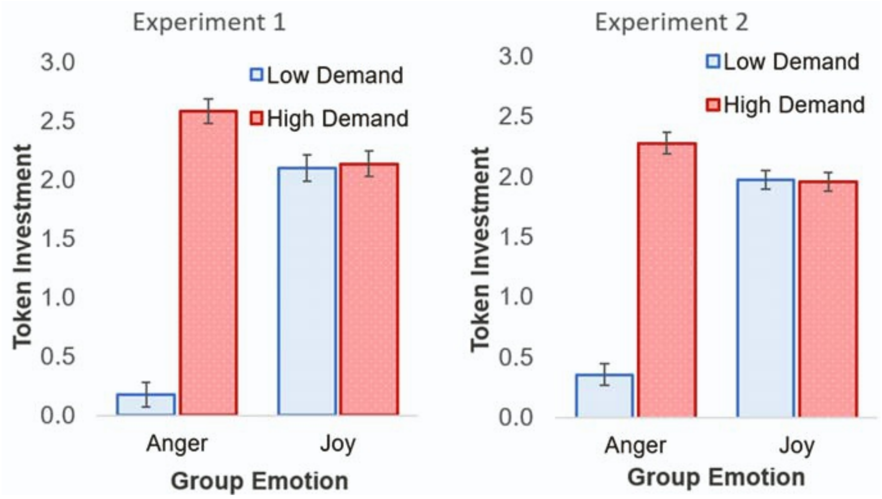

Figure S1. Investment following specific emotions for the low and high demand conditions.

Task Instructions (Experiment 1)

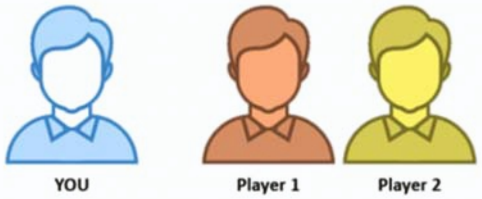

1. Each player starts with an initial endowment of 40 tokens
2. The game lasts for 10 rounds
3. Each player can add 0 to 4 tokens to a public account in each round
4. If in the end the public account contains 60 tokens, everyone keeps their remaining tokens
5. Otherwise, everyone risks losing all their tokens

At the beginning of the experiment you will be randomly assigned to a group, which will include **2 other randomly selected participants**. During the whole experiment, you will interact only with those 2 other group members.

At the beginning of the experiment you and each other group member will receive a personal endowment of **40 tokens**.

The whole experiment consists of **10 rounds** of the following game.

In each round of the game, you have to decide whether to add **0, 1, 2, 3 or 4** tokens in a public account.

If the public account contains at least **60 tokens** after the 10th round, each member of your group will keep their savings, i.e. the tokens of your endowment that were not put in the public account.

Financial incentive

The tokens you keep at the end of the game will be converted to $0.1 and paid through Mechanical Turk as an additional incentive. For example, if you keep 40 tokens at the end of the game, there are at least 60 tokens in the public account, and still win the dice, you will earn $4 in addition to your participation reward of $2.5.

Figure S2. Task instructions for the CRD task.

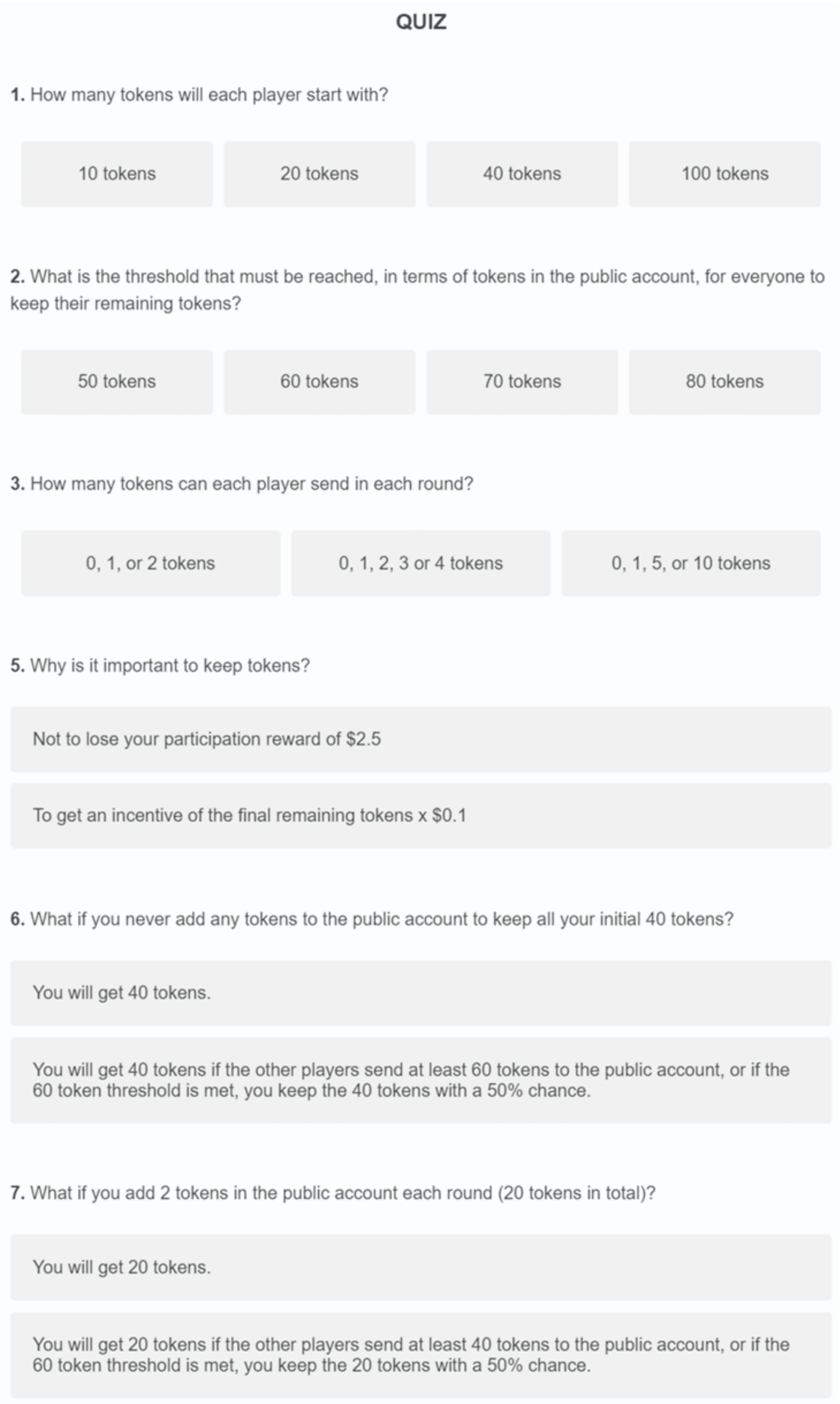

QUIZ

1. How many tokens will each player start with?

- 10 tokens
- 20 tokens
- 40 tokens
- 100 tokens

2. What is the threshold that must be reached, in terms of tokens in the public account, for everyone to keep their remaining tokens?

- 50 tokens
- 60 tokens
- 70 tokens
- 80 tokens

3. How many tokens can each player send in each round?

- 0, 1, or 2 tokens
- 0, 1, 2, 3 or 4 tokens
- 0, 1, 5, or 10 tokens

5. Why is it important to keep tokens?

- Not to lose your participation reward of $2.5
- To get an incentive of the final remaining tokens x $0.1

6. What if you never add any tokens to the public account to keep all your initial 40 tokens?

- You will get 40 tokens.
- You will get 40 tokens if the other players send at least 60 tokens to the public account, or if the 60 token threshold is met, you keep the 40 tokens with a 50% chance.

7. What if you add 2 tokens in the public account each round (20 tokens in total)?

- You will get 20 tokens.
- You will get 20 tokens if the other players send at least 40 tokens to the public account, or if the 60 token threshold is met, you keep the 20 tokens with a 50% chance.

Figure S3. Quiz following the task instructions.

Table S1. Principal component analysis of subjective scales. Rotation method was varimax with Kaiser Normalization.

	Demanding	Fair	Positive
The group often expected me to offer more.	.810	-.300	-.059
The group wished I offered more.	.789	-.081	-.180
The group wanted me to offer a lot of tokens.	.770	-.266	-.032
The group was very demanding.	.761	-.126	-.141
The group felt like I was breaking their social norms.	.665	.269	-.241
The group punished selfish behavior.	.653	.282	-.027
The group felt I was not being fair.	.595	.052	-.398
The group was not very ambitious with respect to my investments.	.557	-.469	.156
The group was very concerned with fairness.	.150	.791	.157
The group wanted all to contribute their fair share.	.132	.771	.181
Were the group's actions, in general, Competitive or Cooperative?	-.257	.700	.406
Were the group's emotions, in general, Competitive or Cooperative?	-.312	.655	.481
The group was happy with my offers.	-.059	.154	.849
The group thought my offers were fair.	-.055	.219	.757
Was the group Hostile or Friendly?	-.275	.551	.592
Was the group Cold or Warm?	-.214	.589	.589
Was the group Pessimistic or Optimistic?	-.307	.529	.568
